# Effective population size does not predict codon usage bias in mammals

**DOI:** 10.1002/ece3.1249

**Published:** 2014-09-23

**Authors:** Michael D Kessler, Matthew D Dean

**Affiliations:** Molecular and Computational Biology, University of Southern California1050 Childs Way, Los Angeles, California, 90089

**Keywords:** Codon usage bias, effective population size, selection

## Abstract

Synonymous codons are not used at equal frequency throughout the genome, a phenomenon termed codon usage bias (CUB). It is often assumed that interspecific variation in the intensity of CUB is related to species differences in effective population sizes (*N*_e_), with selection on CUB operating less efficiently in species with small *N*_e_. Here, we specifically ask whether variation in *N*_e_ predicts differences in CUB in mammals and report two main findings. First, across 41 mammalian genomes, CUB was not correlated with two indirect proxies of *N*_e_ (body mass and generation time), even though there was statistically significant evidence of selection shaping CUB across all species. Interestingly, autosomal genes showed higher codon usage bias compared to X-linked genes, and high-recombination genes showed higher codon usage bias compared to low recombination genes, suggesting intraspecific variation in *N*_e_ predicts variation in CUB. Second, across six mammalian species with genetic estimates of *N*_e_ (human, chimpanzee, rabbit, and three mouse species: *Mus musculus, M. domesticus,* and *M. castaneus*), *N*_e_ and CUB were weakly and inconsistently correlated. At least in mammals, interspecific divergence in *N*_e_ does not strongly predict variation in CUB. One hypothesis is that each species responds to a unique distribution of selection coefficients, confounding any straightforward link between *N*_e_ and CUB.

## Introduction

In most organisms, synonymous codons are not used at equal frequencies. This phenomenon has been termed codon usage bias (CUB), and many studies support a role of natural selection in this phenomenon (Shields et al. 1988; Moriyama and Hartl 1993; Akashi et al. 1998; Comeron and Kreitman 1998; Chamary et al. 2006; Plotkin and Kudla 2011; Waldman et al. 2011; Behura et al. 2013; Kober and Pogson 2013). Proposed mechanisms influencing CUB include translational efficiency (Grantham et al. 1981; Ikemura 1985; Bulmer 1991; Carlini and Stephan 2003; Rocha 2004; Stoletzki and Eyre-Walker 2007; Parmley and Huynen 2009; Hense 2010; Ran and Higgs 2010, 2012; Sharp et al. 2010; Behura and Severson 2011; Shah and Gilchrist 2011; Qian et al. 2012; Agashe et al. 2013; Lawrie et al. 2013; Michely 2013), mRNA stability or folding (Moriyama and Powell 1998; dos Reis et al. 2004; Chamary and Hurst 2005; Chamary et al. 2006; Novoa and Ribas de Pouplana 2012; Kober and Pogson 2013; Shabalina et al. 2013), transcription factor binding (Stergachis 2013), overlap with other functional elements in the genome (Lin 2011), and/or a trade-off between rapid versus accurate translation (Yang et al. 2014).

The level of CUB varies dramatically across species (Grantham et al. 1980a,b; Sharp 1988), including insects (Vicario et al. 2007), mammals (Doherty and McInerney 2013), and plants (Ingvarsson 2008, 2010). Given the large number of codons affecting most cellular processes, the selective benefit associated with any single “preferred” codon should be small. Therefore, CUB is likely to be under weak selection (Akashi 1995; Maside et al. 2004; Cutter and Charlesworth 2006; Haddrill et al. 2010), the efficacy of which will depend on a species’ effective population size (*N*_e_; Kimura 1983; Charlesworth 2009). Consequently, it is often assumed that interspecific variation in CUB can be attributed to interspecific variation in *N*_e_.

Consistent with this hypothesis, *Drosophila simulans* has relatively high CUB compared to *Drosophila melanogaster* (Akashi 1996; McVean and Vieira 2001; Andolfatto et al. 2011) and *D. simulans* has relatively large *N*_e_ (Aquadro et al. 1988). Similarly, *Drosophila pseudoobscura* shows higher codon usage bias than *Drosophila miranda*, with evidence that the former has a larger *N*_e_ (Bachtrog 2007; Haddrill et al. 2010). Outcrossing plant species have more biased codon usage than self-fertilizing relatives (Qiu et al. 2011b), consistent with an expected reduction of *N*_e_ upon the evolution of selfing. However, humans have experienced more evolutionary constraint on codon usage compared to mice (Eory et al. 2010), in spite of their smaller *N*_e_ (Zhao 2000). Thus, it remains unclear whether variation in CUB can be attributed to differences in effective population sizes, especially in mammals.

The hypothesized link between *N*_e_ and CUB assumes that the strength of selection is both small and homogeneous across species. Multiple studies have demonstrated that weak selection shapes patterns of CUB across mammals, including humans, the mammal with the smallest known historical *N*_e_ (Urrutia and Hurst 2003; Comeron 2004; Lu and Wu 2005; Kondrashov et al. 2006; Yang and Nielsen 2008; Waldman et al. 2011; Doherty and McInerney 2013), but see (Urrutia and Hurst 2001; Duret 2002). However, theoretical and empirical studies suggest that selective coefficients may not be homogeneous across species. For example, mutations toward suboptimal codons may be multiplicatively deleterious, so that the strength of selection acting against a suboptimal codon depends on the number of suboptimal codons already present (Kondrashov et al. 2006; Charlesworth 2013). If true, then species with small *N*_e_ may harbor more suboptimal codons, but this may lead to relatively stronger selection against them (Akashi 1995; Hershberg and Petrov 2008), potentially confounding any predicted relationship between *N*_e_ and CUB.

The goal of this manuscript is to test whether *N*_e_ and CUB are correlated in mammals. After demonstrating that codon usage is affected by selection across mammals, we address this goal in two main steps. First, we quantify CUB from 41 mammalian genomes and demonstrate that the observed variation in CUB is not phylogenetically correlated with two indirect proxies for *N*_e_ (age at sexual maturity and body mass). Second, we test for the same correlation across six mammalian species (human, chimpanzee, rabbit, and three mice: *Mus musculus, M. domesticus,* and *M. castaneus*), for which genetic estimates of *N*_e_ existed in the literature, ranging from ∼10K (humans) to ∼780K (rabbits). Although *N*_e_ and CUB are phylogenetically correlated in these latter six species, the effects are modest and inconsistent. At least in mammals, therefore, differences in *N*_e_ do not seem to account for the broad interspecific variation in CUB. One hypothesis is that the distribution of selection coefficients varies across species independently of *N*_e_, confounding any straightforward link to CUB. Direct estimates of selection coefficients using divergence data in two independent species pairs supported this hypothesis.

## Materials and Methods

### Genomes

In the 41-species analyses, all transcripts (exons and introns) were downloaded from Ensembl version 74 (http://www.ensembl.org). For genes with more than one transcript, we chose a transcript randomly for analyses. Across all genes, we repeated the random choice five times, then averaged across the five iterations. CUB is correlated with gene length (Eyre-Walker 1996; Moriyama and Powell 1998; Zeng and Charlesworth 2009), so we also repeated our analyses after systematically choosing the shortest transcript or the longest transcript from all genes. Results from the five random, shortest, and longest transcripts were qualitatively similar; we report the average of the five random choices for simplicity. Only transcripts with at least 100 codons were included due to uncertainty in estimating codon usage in shorter genes (Moriyama and Powell 1998; Novembre 2002).

In the six-species analyses, our aim was to compare only homologous codons. In addition to confining the analysis to one-to-one orthologs between six species, we excluded all codons that had an ambiguity or indel in any one species, as well as all codons that were 3′ to the earliest stop codon of any one species (Appendix S1). We dealt with multiple transcripts as described above. Complete genomes of *M. domesticus*, *M. musculus*, and *M. castaneus* were downloaded from Keane et al. (2011), then transcripts and flanking regions were computationally assembled using the coordinates of the mouse genome annotations of Ensembl version 65 (http://www.ensembl.org). Due to incomplete lineage sorting and/or hybridization, phylogenetic relationships among these three mouse species vary across the genome (White et al. 2009); we therefore added the three mouse species to the phylogeny as an unresolved trichotomy with a common ancestor 350K years ago (Geraldes 2008). One-to-one orthologs among species were identified using the phylogenetic analyses of Ensembl version 65: approximately 11,000 genes had one-to-one orthologs across all six species and a minimum of 100 codons of alignment. We translated each set of transcripts into proteins, aligned protein sequences with both PRANK (Löytynoja and Goldman 2005) and CLUSTALW (Thompson et al. 1994), and then back-translated aligned sequences to their original DNA sequences. We report results based on PRANK-aligned sequences; our conclusions did not change if we used CLUSTALW-aligned sequences.

### Quantifying CUB

There are multiple methods for quantifying codon usage bias. The “effective number of codons” (ENC; Wright 1990) and variations thereof (Fuglsang 2006) quantify deviation from the null hypothesis that synonymous codons within each amino acid class are used at equal frequency. However, that null hypothesis assumes equal frequency of the four nucleotides, which could be violated if the mutational process is biased (Palidwor et al. 2010; Zhang 2012) or if base composition varies across the genome (Bernardi 1995, 2000). To control for biased mutational processes, Novembre (2002) proposed the “effective number of codons Prime” (ENCp), deriving expected codon usage from local base composition. If the four bases are equally frequent, ENCp reduces to ENC. Both ENC and ENCp theoretically range from 20 (every amino acid coded by a single codon, representing maximal bias) to 61 (each amino acid coded by each of its synonymous codons at equal frequency, representing minimal bias). We calculated ENC and ENCp using Novembre's ENCprime software, which quantifies the significance of observed versus expected codon usage via Pearson's *χ*^2^ statistics (Novembre 2002). Alternative methods, such as the “frequency of preferred codons” (Ikemura 1981) or the “codon adaptation index” (Sharp and Li 1987; Lee et al. 2010), require a priori definition of “preferred codons,” which may not be conserved across species (Hershberg and Petrov 2009; Rao 2011). Furthermore, selection may favor an overall balanced combination of preferred and unpreferred codons at the genomic scale so that genes vary in their preferred and unpreferred codons (Shah and Gilchrist 2011; Qian et al. 2012; Agashe et al. 2013; Yang et al. 2014). All results presented below were qualitatively similar whether we use ENC or ENCp; we report ENCp.

When estimating ENCp, we estimated the background base composition using either the 2 kb flanking sequence on each side of the gene (4 kb total) or the concatenated introns of each transcript. The latter approach controls for mutational processes specifically related to transcription; however, many genes drop out of the analysis because they did not meet our minimum requirement of having at least 1000 bp of intronic DNA. Both strategies yielded nearly identical results (Appendix S2); we report results based on flanking regions.

### Testing for selection

One of the primary assumptions behind the hypothesis that CUB scales with *N*_e_ is that codon usage is shaped by selection. In addition to existing literature on the subject (see Introduction), we tested for selection using three main methods. First, we quantified the number of genes that showed ENCp significantly different than expectations built from local base composition across the 41 mammalian species (Novembre 2002).

Second, we implemented the methodology of Yang and Nielsen (2008) to specifically test whether codon usage was influenced by selection across the six mammalian species for which we had independent genetic estimates of effective population size. Under a FMutSel0 model, codon usage evolves only by mutational bias and is unaffected by selection. Under a FMutSel model, synonymous mutations can fix according to differences in fitness between synonymous codons. A likelihood ratio test (LRT), quantified as twice the difference in log-likelihoods of the two models, distributed as a *χ*^2^ distribution with degrees of freedom equal to the difference in the number of parameters estimated, is a formal test of whether selection affects codon usage.

Third, we tested for selection with a novel approach that focused on resequencing data from humans, the species with the smallest *N*_e_, and therefore the least likely to be affected by selection. We analyzed the 1000 genome data (Consortium TGP 2012) from the Yoruban population in order to minimize effects of known bottlenecks in non-African populations. For amino acids with redundant codons (sixfold redundant amino acids were divided into their respective fourfold and twofold redundant classes, Rocha 2004; Sun et al. 2013), we considered the most frequently used codon in the genome as “preferred” and the least frequently used codon as “unpreferred”. Inaccuracy in defining preferred/unpreferred codons in this way should only add noise to our analysis, making our conclusions below conservative. A McDonald–Kreitman framework (McDonald and Kreitman 1991) was then applied to test whether the ratio of fixed: polymorphic sites differed between unpreferred-to-preferred: preferred-to-unpreferred mutations, with polarity determined by comparison to the chimp + gorilla genomes. To generate null expectations and account for possible mutational biases that could mimic codon bias, we repeated the analysis in introns, forcing segregating sites to be a third position in an imaginary codon. Intronic “codons” from reverse-transcribed genes were also reverse complemented. Any segregating sites in an intron that fell within 20 bp of an exon–intron boundary were excluded. For all codons, we also gathered the +1 and +2 position so that we could repeat the analyses after excluding sites that could have been mis-polarized due to CpG hypermutability on either strand. Specifically, an XXTGX in chimps + gorilla to XXCGX mutation in human could be falsely polarized if two independent CpG->TpG mutations occurred in chimp and gorilla (X indicates any base with the constraint that they are the same across species; 5′ to 3′ of coding direction is shown, with the 3rd codon position in the middle of the quintet). By similar logic, XCAXX in chimp + gorilla and XCGXX in humans could arise via two independent cytosine deaminations on the other strand.

### Quantifying *N*_e_

For the 41-species analyses, robust estimates of *N*_e_ do not exist for most species, so we turned to two indirect proxies of *N*_e_ – body mass and age at sexual maturity – gleaned from the literature (Appendix S3). Large and/or slowly reproducing mammals tend to have small population sizes (Ohta 1972; Damuth 1981).

For the six-species analyses, genetic estimates of *N*_e_ were taken from the literature: human (Zhao 2000), chimp (Won and Hey 2005), rabbit (Carneiro et al. 2009), and the three mouse species (Geraldes 2008; Geraldes et al. 2011). We confined our analyses to those studies for which *N*_e_ was estimated from resequencing data rather than genotyping known polymorphisms because the latter strategy suffers from ascertainment bias. Although the studies cited obviously differ in methodology, they were largely drawn from noncoding regions of the genome, to avoid assaying regions affected by selection. Estimates of *N*_e_ ranged roughly 78-fold, from ∼10K in humans to ∼780K in rabbits.

We tested the correlation between *N*_e_ (or its proxies) and ENCp using the gls procedure in the R package nlme, with a correlation structure that accounted for phylogenetic relatedness (Pagel 1999), built with the corPagel procedure in the R package ape (Paradis et al. 2004). We used the phylogenetic relationships and branch lengths inferred by Meredith et al. (2011). In the 6-species analyses, convergence was unstable; we therefore repeated each gls under all 6! = 720 unique orders in which taxa could be input into the analysis and report median statistical values (Appendix S4).

We repeated the analyses after dividing genes into groups expected to show intraspecific variation in *N*_e_. For species whose public genomes included chromosomal compartment, we tested whether ENCp differed among autosomal versus X-linked genes. Assuming an equal effective sex ratio (which may not be a valid assumption, Hammer et al. 2008; Hammer 2010), the X chromosome has an *N*_e_ predicted to be three-fourths as large as each autosome. Positive selection on X-linked recessives will further reduce the effective population size of the X chromosome, due to selection at linked sites (Maynard Smith and Haigh 1974; Kaplan et al. 1989; Andolfatto and Przeworski 2001; Kousathanas et al. 2014).

Similar to X-linked versus autosomal comparisons, genes in regions of low recombination should have reduced *N*_e_, because they are more likely to be in physical linkage with sites under selection (Hill and Robertson 1966). Consistent with this intuition, codon usage was reduced in regions with relatively low recombination in *Drosophila* (Kliman and Hey 1993; Hey and Kliman 2002; Marais and Piganeau 2002). Because recombination rates are not known for all species, we considered genes within 10 MB of the centromere boundary to be in relatively low recombination regions compared to genes within 10 MB of the telomere boundary. In mouse (human), this demarcation was biologically relevant, where the average recombination rate was 0.43 cM/Mb (4.6 cM/Mb) for centromeric regions and 0.58 cM/Mb (8.7 cM/Mb) for telomeric regions; Wilcoxon rank-sum test *P* = 0.015 (*P* = 10^−10^). Mouse recombination maps were taken from Cox (2009), while human recombination maps were downloaded from UCSC Genome Browser's HapMap2 for the GRCh37 (HG19) build.

To test for heterogeneity across different classes of genes or sites, we repeated all analyses for (1) the bottom, top, and middle third of genes ranked by ENCp; (2) each amino acid group separately (e.g., Kliman 2014; Yang et al. 2014); and (3) after excluding potential exon splice enhancers. For this latter analysis, we excluded the first 15–17 bases (five codons plus 0–2 additional base pairs to preserve reading frame) from each end of every coding exon from each transcript. Such regions may be constrained to act as exon splice enhancer elements (Eyre-Walker and Bulmer 1993; Parmley and Hurst 2007; Warnecke and Hurst 2007; Gu et al. 2010; Lin 2011) and may experience less efficient selection compared to internal codons (Loewe and Charlesworth 2007).

## Results

### Codon usage bias is shaped by selection in mammals

Using three different approaches, we found strong evidence that CUB has been shaped by selection across mammals. First, across the 41 mammalian genomes, an average of 91.1% of genes (range: 85.8–93.7%; average number of 17,271 genes analyzed, range: 10,235–19,410) showed significant evidence of selection (ENCp more biased than expected at *P* < 0.05 after Benjamini–Hochberg correction).

Second, for the six-species analysis, approximately 90% of the 11,000 orthologous gene alignments showed statistically significant evidence of selection (twice the difference in log-likelihoods estimated for the FMutSel0 versus FMutSel models ≥ 56.94, df = 41, *P* < 0.05, significance determined after Benjamini–Hochberg correction, Yang and Nielsen 2008). This number is similar to Yang and Nielsen (2008), who found evidence of selection in 94% of genes evolving across a phylogeny of five divergent mammal species. We repeated the analysis using only human and chimp, the two species with the smallest effective population sizes. Our power to detect selection is expected to plummet for the human–chimp comparison after trimming out most of the evolutionary divergence from the phylogeny. Furthermore, we might expect to detect less selection given these are the two species with the smallest effective population sizes. In spite of these two expected limitations, we still found statistically significant evidence for selection in 77% of orthologous genes (likelihood ratio test ≥ 56.94, df = 41, *P* < 0.05, significance determined after Benjamini–Hochberg correction). This number is similar to the 87% of genes found to be under selection by Yang and Nielsen (2008), who compared human and macaque.

Third, Yoruban genomes showed a significantly higher proportion of variable sites that are fixed for unpreferred-to-preferred versus preferred-to-unpreferred mutations (0.49 vs. 0.43, *χ*^2^ = 66.62, *P* < 10^−15^, Table1). Interestingly, this same pattern was observed in fake “codons” constructed from intronic regions (0.36 vs. 0.35, *χ*^2^ = 458.4, *P* < 10^−15^, Table1), suggesting some mutational bias mimics some patterns of codon usage bias. However, the observed *χ*^2^ deviation normalized by the total number of observed mutations was more than an order of magnitude larger in exonic versus intronic regions (*χ*^2^/*N* = 2.60 vs. 0.23, respectively, Table1), strongly suggesting that selection favors unpreferred-to-preferred mutations in exons above and beyond mutational biases. After excluding any sites that could have arisen via CpG hypermutation, the overall patterns remain the same (numbers in parentheses of Table1).

**Table 1:** Evidence that selection affects codon usage bias in humans. Before (After) controlling for potential CpG mis-polarization.

	Polymorphic	Fixed^1^	*P* (Fixed)	Chisq	*P*	Chisq/*N*
Exons						
Preferred→unpreferred	10,249 (10,249)	7729 (7729)	0.43 (0.43)			
Unpreferred→preferred	3957 (2859)	3731 (2487)	0.49 (0.47)	66.62 (20.71)	10^−15^ (10^−5^)	2.60 (0.89)
Intron						
Preferred→unpreferred	690,248 (690,248)	366,993 (366,993)	0.35 (0.35)			
Unpreferred→preferred	609,530 (521,822)	345,218 (285,076)	0.36 (0.35)	458.46 (76.7)	10^−15^ (10^−15^)	0.23 (0.04)

1 Polarized by comparison of human segregating sites to chimpanzee + gorilla genomes.

In sum, all three approaches provided strong support that selection has shaped codon bias in mammals, even those species with the smallest effective population sizes. Our results are consistent with a growing body of work demonstrating codon usage is under selection in mammals (Urrutia and Hurst 2003; Comeron 2004; Lu and Wu 2005; Kondrashov et al. 2006; Yang and Nielsen 2008; Waldman et al. 2011; Doherty and McInerney 2013). Other studies have argued that patterns of selection in mammals are either absent or the result of mutational processes or methodological artifacts (Urrutia and Hurst 2001; Duret 2002). These latter studies point out the confounding factors that mutational processes and base composition have on estimates of codon usage bias, which are controlled for in all three approaches used above.

### Codon usage bias was not correlated to inferred variation in *N*_e_ across 41 mammalian species

**Figure 1:**
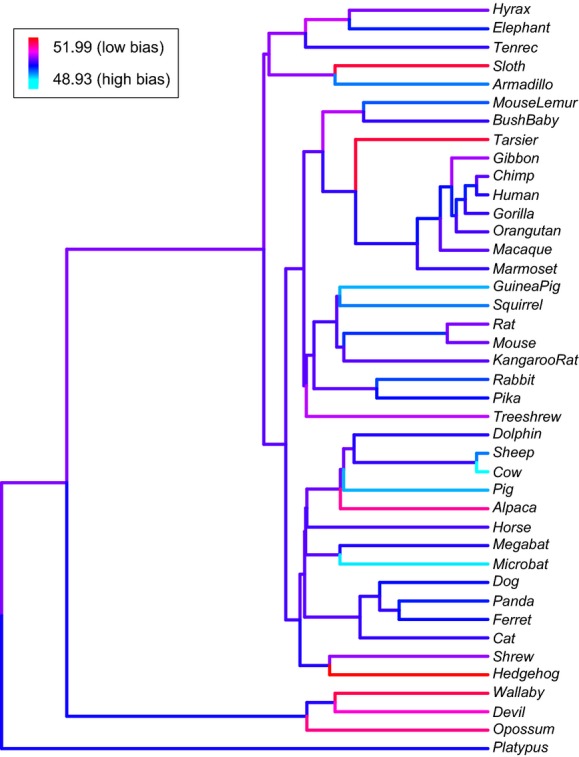
Median genome-wide effective ENCp estimated across 41 mammalian species. Values of ENCp were estimated on internal branches using the maximum-likelihood ace function in the ape package of R (Paradis et al. 2004).

**Figure 2:**
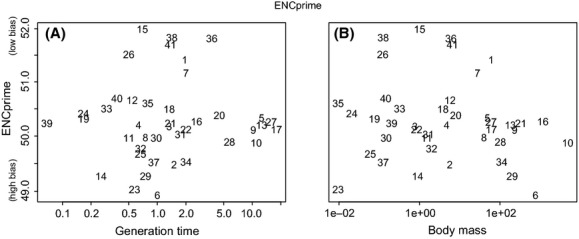
Between species, log effective number of codons Prime (ENCp) did not correlate with two different proxies of species’ effective population sizes: (A) log_10_ generation time or (B) log_10_ body mass. Taxa numbered alphabetically: 1-Alpaca, 2-Armadillo, 3-Bush baby, 4-Cat, 5-Chimp, 6-Cow, 7-(Tasmanian) Devil, 8-Dog, 9-Dolphin, 10-Elephant, 11-Ferret, 12-Gibbon, 13-Gorilla, 14-Guinea Pig, 15-Hedgehog, 16-Horse, 17-Human, 18-Hyrax, 19-Kangaroo Rat, 20-Macaque, 21-Marmoset, 22-Megabat, 23-Microbat, 24-Mouse, 25-Mouse Lemur, 26-Opossum, 27-Orangutan, 28-Panda, 29-Pig, 30-Pika, 31-Platypus, 32-Rabbit, 33-Rat, 34-Sheep, 35-Shrew, 36-Sloth, 37-Squirrel, 38-Tarsier, 39-Tenrec, 40-Treeshrew, 41-Wallaby.

In spite of the evidence that selection shapes codon usage, ENCp was not correlated to either proxy of effective population size. ENCp varied from the most biased score of 48.93 in cow to the least biased score of 51.99 in hedgehog (Fig.1, Appendix S3). Variation in log_10_ ENCp was not correlated to log_10_ age at sexual maturity (phylogenetically controlled *t*_39_ = −0.09, *P* = 0.93, Fig.2A) or log_10_ body mass (phylogenetically controlled *t*_39_ = −1.35, *P* = 0.18, Fig.2B). For the most part, we did not find either correlation if we analyzed (1) the lowest, intermediate, or highest third of genes ranked according to ENCp (generation time: *t*_39_ = −0.44, −0.08, 1.15; *r* = −0.19, −0.14, −0.02; *P* = 0.67, 0.93, 0.26, for the three groups, respectively; body mass: *t*_39_ = −0.99, −1.33, −0.39; *r* = −0.21, −0.27, −0.09; *P* = 0.33, 0.19, 0.70, for the three groups, respectively); (2) each amino acid family separately (exceptions being twofold redundant arginine where ENCp correlated to generation time and threonine where ENCp correlated to body mass, Appendix S5); or (3) each transcript after excluding potential exon splice enhancers (correlation to log_10_ generation time: *t*_39_ = −0.09, *r* = −0.16, *P* = 0.92; correlation to log_10_ body mass: *t*_39_ = −1.24, *r* = −0.30, *P* = 0.22).

The phylogenetically controlled methods just presented test for a linear relationship between ENCp and *N*_e_. To check for nonlinear relationships, we performed a simple nonparametric test, asking whether codon usage bias tended to increase in those parts of the phylogeny where effective population size increased, regardless of magnitude. After calculating phylogenetically independent contrasts using the pic function in ape (Paradis et al. 2004), there was no evidence for this pattern using either proxy of effective population size (for both body mass and generation time: 22 of 40 independent contrasts showed increased codon bias with increased *N*_e_, Fisher's exact test *P* > 0.65), again arguing against a strong correlation between codon usage and effective population size.

One possible explanation for the lack of a strong correlation between *N*_e_ and CUB is that each species is subject to its own unique distribution of selection coefficients associated with codon usage. If true, then within each species, variation in CUB may still correlate with intragenomic variation in *N*_e_. To test this prediction, we now turn our attention to comparisons of genes that are X-linked versus autosomal, as well as in high versus low recombination regions.

### Codon bias was weaker for X-linked genes

**Figure 3:**
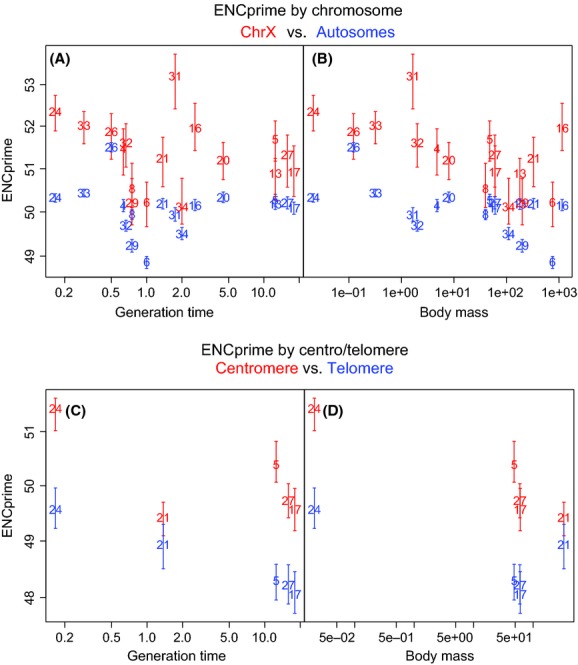
Within species, ENCp differed across genomic compartments predicted to have different effective population sizes: X versus autosomes (A and B) or centromeric versus telomeric regions (C and D). Species numbered as in Figure2. Bars represent 95% confidence intervals of medians estimated by bootstrapping the dataset 10,000 times.

For all 17 species for which chromosomal compartment was annotated, autosomal genes were more biased than X-linked genes, significantly so for 15/17 species (*P* < 0.05 after Benjamini–Hochberg correction, the exceptions being gorilla and opossum), in an analysis of covariance (ANCOVA) taking into account the important covariates of exon and intron lengths (Moriyama and Powell 1998; Duret and Mouchiroud 1999; Comeron and Kreitman 2000; Vinogradov 2001; Stoletzki and Eyre-Walker 2007; Stoletzki 2011; Behura et al. 2013; Fig.3A and B, Appendices S3 and S6). The reduced bias of X-linked genes is consistent with their expected reduction in *N*_e_ relative to autosomes, although much of the variation remains to be explained.

The pattern of reduced CUB on X-linked genes observed here is opposite that observed in flies, worms, and plants, where X-linked genes were more strongly biased than autosomes (Singh et al. 2005; Haddrill et al. 2010; Zeng and Charlesworth 2010; Qiu et al. 2011a). Although outside the main focus of this manuscript, the differences may be due to differences in X inactivation, dosage compensation, and/or the history of gene traffic between X and autosomes (Emerson et al. 2004).

### Codon bias was weaker for low recombination genes

For all five species for which centromere and telomere were annotated, centromeric genes were less biased then telomeric genes, significantly so for 4/5 species (*P* < 0.05 after Benjamini–Hochberg correction, with marmoset the exception) in an ANCOVA taking into account length of exons and introns (Fig.3C and D, Appendices S3 and S7). As with comparisons between X chromosomes and autosomes, this result is consistent with the idea that intragenomic differences in *N*_e_ predict variation in CUB. Although recombination itself may favor mutations toward GC (Marais et al. 2001), such processes are not expected to explain our results because ENCp takes mutational biases into account. As argued in Materials and Methods, we chose a biologically meaningful chromosome length (10 Mb) to define centromeric and telomeric loci, but we uncovered the same qualitative results if used either 20 Mb or 5 Mb cutoffs instead.

### Codon usage bias was not strongly correlated to variation in genetic estimates of *N*_e_ across six mammalian species

Among the six mammalian species for which independent genetic estimates of *N*_e_ existed, there was a significant correlation between *N*_e_ and ENCp (phylogenetically controlled *t* = −5.716, *r* = −0.617, *P* = 0.005, Appendix S8). When analyzed separately, 18.1% of genes showed a significant, negative correlation between *N*_e_ and ENCp (*P* < 0.05 after Benjamini–Hochberg correction). However, the differences in ENCp were modest, ranging from 51.41 in humans to 50.46 in rabbit even though *N*_e_ varies by roughly 78-fold among these species (Appendices S8 and S9). Furthermore, 15.0% of genes showed a significant, *positive* correlation between *N*_e_ and ENCp (*P* < 0.05 after Benjamini–Hochberg correction), opposite the prediction of weak selection. Although rabbit is clearly an outlier (Appendix S8), removing it did not change the significance (phylogenetically controlled *t* = −2.892, *r* = −0.5992, *P* = 0.01).

For the six-species analyses, three different subsets of the data revealed some interesting patterns. First, for genes ranked in the lowest third of ENCp (indicating high bias), there was not a statistically significant correlation to *N*_e_ (phylogenetically controlled *t*_4_ = 2.32, *r* = 0.028, *P* = 0.08 [although there is a trend, note that the positive *t*-value indicates it is in the opposite direction predicted by weak selection]). The relationship held for the other two groups (phylogenetically controlled *t*_4_ = −3557340, −16.46; *r* = −0.53, −0.82; *P* ≤ 0.0001, 0.0001, for the intermediate and highest ranked thirds, respectively). Second, when we analyzed each amino acid family separately, 18 of the 21 amino acid groups showed statistically significant evidence of the correlation (phylogenetically controlled *P* < 0.05 in all cases, the exceptions being asparagine, the fourfold serine, and the twofold leucine, Appendix S10). However, two of the 18 significant results (proline and threonine) showed a correlation in the opposite direction predicted by weak selection (Appendix S10). Third, when transcripts were analyzed after removing potential exon splice enhancers, there was not a significant correlation between *N*_e_ and codon usage (phylogenetically controlled *t* = 2.15, *r* = 0.29, *P* = 0.09 [although there is a trend, note that it is in the opposite direction predicted by weak selection]). Removing potential exon splice enhancers removed a median of 252 bp from transcripts, covering a median 21.3% of each transcript. Overall, then, there is not a consistently strong correlation between *N*_e_ and CUB in the 6-species analyses.

## Discussion

It is often assumed that interspecific variation in effective population size (*N*_e_) explains a significant amount of the interspecific variation in CUB. We find almost no support for the correlation between *N*_e_ and CUB. In our 41-species analyses, we did not uncover any evidence that interspecific differences in *N*_e_ predicted variation in codon usage bias. In the six-species analyses, we uncovered a significant phylogenetic correlation, but the differences in CUB were subtle in spite of a 78-fold range in *N*_e_ and inconsistent across different subsets of the data. Furthermore, even though rabbit has by far the largest mammalian *N*_e_ in the present study, it does not have the most biased genome (Fig.1, Appendix 3). On the whole, our study does not support a strong relationship between effective population size and codon usage bias.

There are multiple hypotheses that could explain why *N*_e_ and CUB were not strongly correlated in our study. The general prediction that Ne predicts CUB assumes that the average selective coefficient affecting codon usage is both small and homogenous across species. Although these assumptions seem to hold in a variety of studies (see Introduction), they may be violated under some scenarios. For example, selection associated with CUB may be stronger than previously appreciated (Carlini and Stephan 2003; Lawrie et al. 2013), may vary according to the number of suboptimal codons in a genome (Akashi 1995; Hershberg and Petrov 2008), or may act synergistically (Kondrashov et al. 2006; Charlesworth 2013). Another possibility is that species with large *N*_e_ experience elevated rates of adaptive evolution on protein coding genes, potentially interfering with selection on codon usage (Betancourt and Presgraves 2002; Haddrill et al. 2011; Phifer-Rixey 2012), though such an effect has been argued to be weak (Bierne and Eyre-Walker 2006). Ultimately, codon usage bias is correlated to many factors (Behura and Severson 2013), especially gene expression level (Gouy and Gautier 1982) and it may simply be that the appropriate data for parsing out different forces affecting CUB (e.g., gene expression data across tissues and species) are currently lacking. Species divergence in any of these correlates could obscure any simple link between CUB and *N*_e_. We now explore three potential hypotheses in more detail.

First, the distribution of selection coefficients may differ across species. For example, species with more deleterious codon usage might experience stronger selection toward preferred codons (Akashi 1995; Kondrashov et al. 2006; Hershberg and Petrov 2008; Charlesworth 2013). To test this hypothesis further, we estimated |*N*_e_*s| (Yang and Nielsen 2008), where s is the selective coefficient acting specifically on codon usage, for two independent species pairs: human–chimp and *M. castaneus–M. domesticus*. The median |*N*_e_*s| did not differ across 11,000 genes (median |*N*_e_*s| = 6.61, 6.62 for rodents and primates, respectively; Wilcoxon rank-sum test, *P*-value = 0.93) even though the *N*_e_ of the rodent species pair (*M. castaneus* = 220K, *M. domesticus* = 100K) is more than nine times larger than the *N*_e_ of the primate species pair (chimp = 25K, human = 10K). By extension, the average selection coefficient associated with codon usage must be roughly nine times larger in primates compared to rodents, supporting the hypothesis that the selection coefficients vary across species.

Second, other predictors of the efficacy of selection, such as recombination rate, may differ between species, and could obscure any straightforward predictions about the effects of population size. A growing body of evidence suggests that species with small *N*_e_ have evolved increased rates of recombination. For example, the number of chiasmata is positively correlated with generation time across mammals (Burt and Bell 1987). Additionally, artificial selection experiments, where organisms experience both a bottleneck in numbers and an increase in selective intensity, often result in the evolution of increased recombination rate (Burt and Bell 1987; Otto and Lenormand 2002). Recombination rates for three mouse species studied here (*M. castaneus*, *M. domesticus*, and *M. musculus*) vary by ∼30%; *M. musculus*, the species with the smallest effective population size, has the largest recombination rate while *M. castaneus*, the species with the largest effective population size, has the smallest recombination rate (Dumont et al. 2011). Furthermore, a small island population of *M. domesticus* has evolved a higher recombination rate compared to classic strains of *M. domesticus* (Dumont and Payseur 2011). It is possible that reductions in effective population size are somewhat counterbalanced by the expected increase in selective efficiency gained by elevated rates of recombination, complicating straightforward predictions about *N*_e_-CUB correlations.

Third, codon usage bias may simply evolve on a different time scale than effective population size or its correlates (Jensen and Bachtrog; Marais et al. 2004; Zeng and Charlesworth 2009, 2010), which would be especially important if populations frequently deviate from equilibrium (Zeng and Charlesworth 2009). However, we note that patterns of adaptive protein evolution correlated with effective population size were detected in the three mouse species studied here (Phifer-Rixey 2012), even though they have only been separated for ∼350K years (Geraldes 2008). Thus, the timescale would seem to be long enough to detect a correlation if it existed.

In sum, species-specific selection coefficients and/or recombination rates may obscure the predicted correlation between CUB and *N*_e_ across 41 mammalian species. At least in mammals, our study rejects the common assumption that interspecific differences in codon usage can be attributed to variation in effective population sizes. This pattern may be widespread: across 13 independent pairs of eukaryotic species, Gossmann et al. (2012) failed to find a correlation between CUB and *N*_e_. Gossmann et al. (2012) analyzed two mammalian species pairs, averaging 261 genes; our study extends to 41 mammalian genomes. The continued accumulation of population level resequencing data and whole genomes, as well as independent estimates of *N*_e_ over a broader range of taxa, will shed further light on the evolutionary processes that shape codon usage.

## Supporting Information

**Appendix S1.** In the six-species analysis, only aligned codons that could be analyzed from all species were included. In this hypothetical example, gray boxes indicate codons that would be excluded because: (1) an indel occurs in codon 2 in Species 1; (2) a heterozygous site (or sequence ambiguity) occurs at codon 4 in Species 2; and (3) an early stop codon occurs in Species 4.

**Appendix S2.** Estimates of ENCp using flanking DNA or intronic DNA did not qualitatively change our results. Both methods are highly correlated (*P* < 2e-16, *r*^2^ = 0.86, solid line). Using a 1:1 line as comparison (dashed line), measures of ENCp were generally more biased when we used intronic DNA versus flanking DNA. Species numbered as in Figure 2.

**Appendix S3.** The 41 mammalian genomes analyzed. Average age of sexual maturity and average body mass taken from http://animaldiversity.ummz.umich.edu, last accessed Jan. 2014. 95% confidence intervals of median codon bias estimated by bootstrapping.

**Appendix S4.** Histogram of *P* values across all 6! = 720 possible ways to add 6 taxa to the analysis. Not all permutations yielded the same *P* value, but all would be called statistically significant.

**Appendix S5.** The results of a phylogenetic generalized least squares correlation between ENC (not ENCp) and either generation time or body mass. All codons within an amino acid class were concatenated; because local base composition is nonsensical when combining codons across the genome, these tests were performed from ENC, not ENCp.

**Appendix S6.** Results of ANCOVA's across species, testing the effect of X versus autosomal compartments (Chrom), the total number of coding base pairs (Exon), the total number of base pairs in introns between coding exons (Intron), and their interactions.

**Appendix S7.** Results of ANCOVA's across species, testing the effect of centromeric versus telomeric location (location), the total number of coding base pairs (Exon), the total number of base pairs in introns between coding exons (Intron), and their interactions.

**Appendix S8.** Log_10_ ENCp versus log_10_ *N*_e_ across six mammal species. Bars represent 95% confidence intervals of medians estimated by bootstrapping the dataset 10,000 times.

**Appendix S9.** Point estimates and bootstrap ranges for *N*_e_ and ENCp for the six-species analysis.

**Appendix S10.** The results of a phylogenetic generalized least squares correlation between ENC (not ENCp) and genetic estimates of *N*_e_ from six mammalian species per amino acid. All codons within an amino acid class were concatenated; because local base composition is meaningless when combining codons across the genome, these tests were performed with ENC, not ENCp.
