# Supplementary figures and images for: Effective population size does not predict codon usage bias in mammals
Source: Ecol Evol. 2014 Sep 23;4(20):3887–900. doi: 10.1002/ece3.1249 (PMC4242573; doi:10.1002/ece3.1249)

**Appendix S1.**


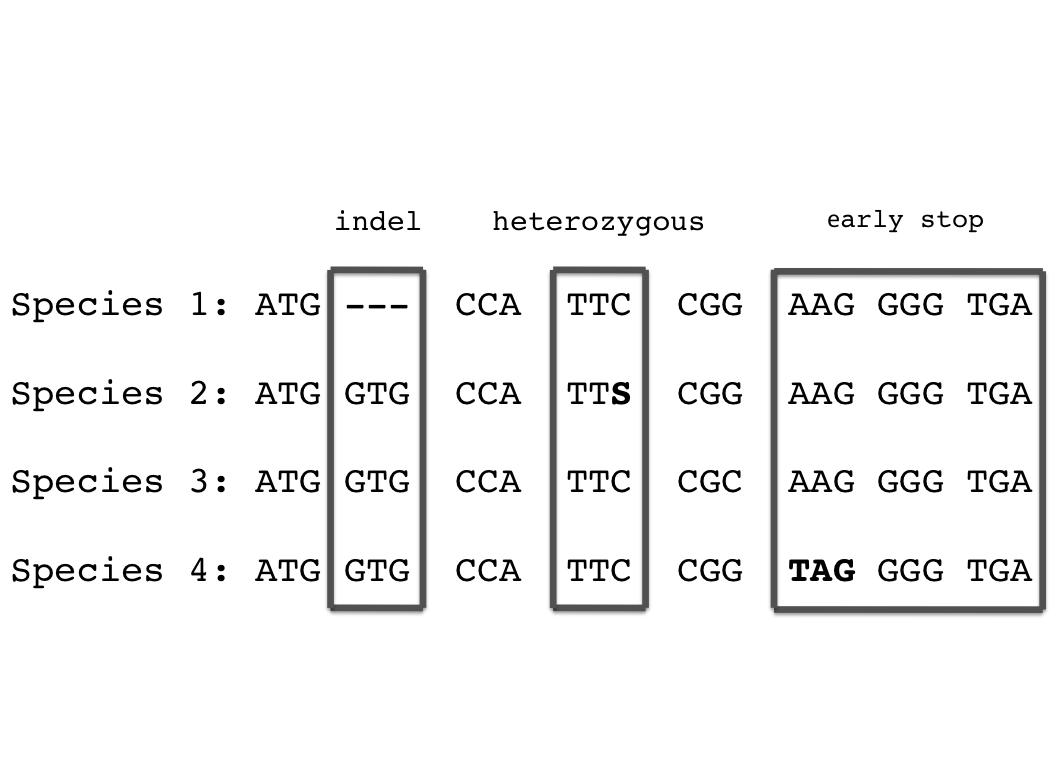

Supplement: Supplementary file 1 — Appendix S1. In the six-species analysis, only aligned codons that could be analyzed from all species were included. In this hypothetical example, gray boxes indicate codons that would be excluded because: (1) an indel occurs in codon 2 in Species 1; (2) a heterozygous site (or sequence ambiguity) occurs at codon 4 in Species 2; and (3) an early stop codon occurs in Species 4. [file ece30004-3887-SD1.docx]

**P values estimated from GLS**

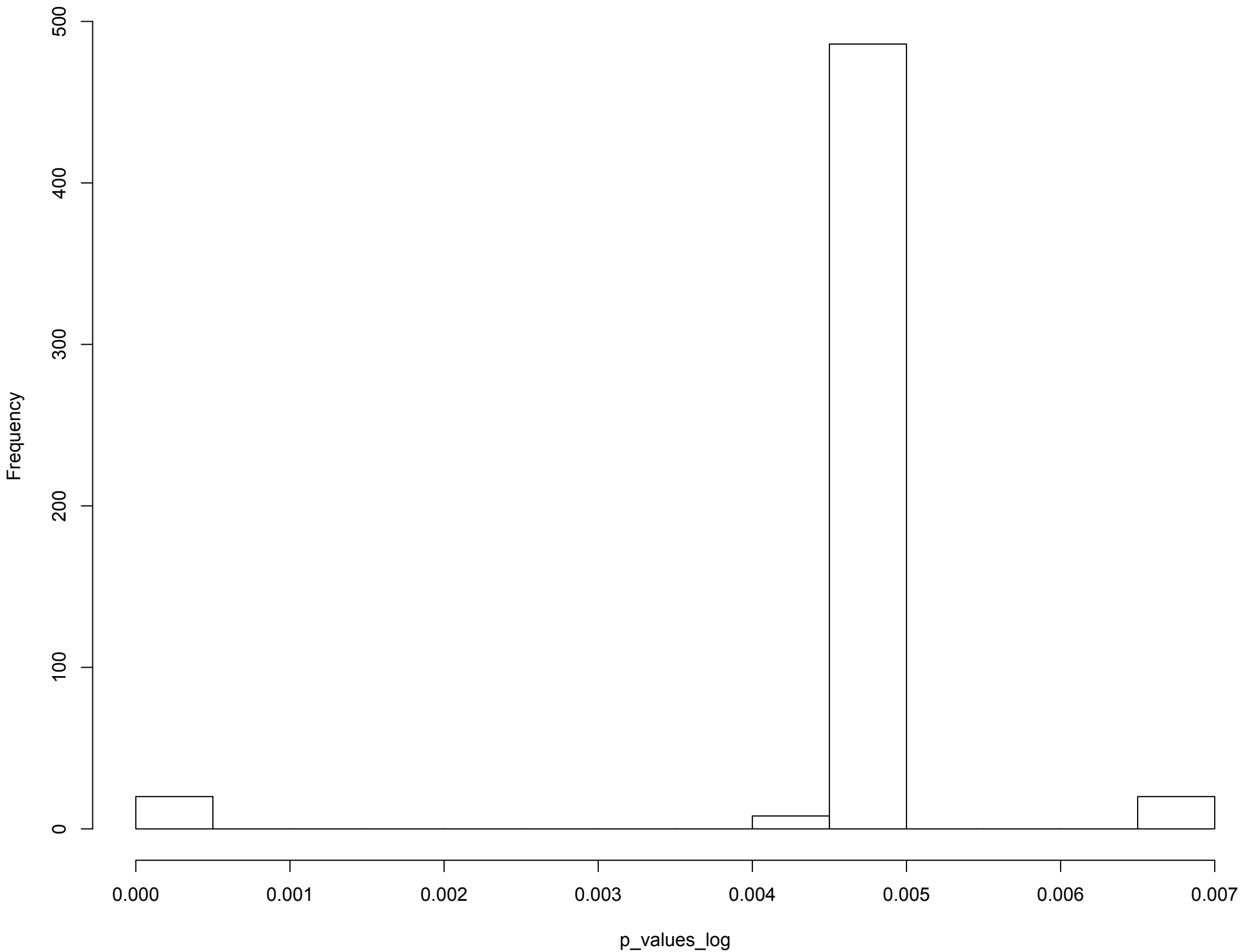

Supplement: Supplementary file 4 — Appendix S4. Histogram of P values across all 6! = 720 possible ways to add 6 taxa to the analysis. Not all permutations yielded the same P value, but all would be called statistically significant. [file ece30004-3887-SD4.pdf]
