# Supplementary material for: Effective population size does not predict codon usage bias in mammals
Source: Ecol Evol. 2014 Sep 23;4(20):3887–900. doi: 10.1002/ece3.1249 (PMC4242573; doi:10.1002/ece3.1249)
Supplement: Supplementary file 2 — Appendix S2. Estimates of ENCp using flanking DNA or intronic DNA did not qualitatively change our results. Both methods are highly correlated (P < 2e-16, r2 = 0.86, solid line). Using a 1:1 line as comparison (dashed line), measures of ENCp were generally more biased when we used intronic DNA versus flanking DNA. Species numbered as in Figure 2. [file ece30004-3887-SD2.docx]

**Appendix S2.**
