# Supplementary material for: Effective population size does not predict codon usage bias in mammals
Source: Ecol Evol. 2014 Sep 23;4(20):3887–900. doi: 10.1002/ece3.1249 (PMC4242573; doi:10.1002/ece3.1249)
Supplement: Supplementary file 3 — Appendix S3. The 41 mammalian genomes analyzed. Average age of sexual maturity and average body mass taken from http://animaldiversity.ummz.umich.edu, last accessed Jan. 2014. 95% confidence intervals of median codon bias estimated by bootstrapping. [file ece30004-3887-SD3.pdf]

Appendix S3. The 41 mammalian genomes analyzed. Average age of sexual maturity and average body mass taken from http://animaldiversity.ummz.umich.edu. 95% confidence intervals of medians estimated by bootstrapping.

CommonName	Species	Average age at sexual maturity (years)	Average body mass (kg)	ENCp (95% CI)	N	ENCp (95% CI), chrX	N	ENCp (95% CI), autosomes	N	ENCp (95% CI), telomere	N	ENCp (95% CI), centromere	N
Alpaca	Vicugna pacos	1.94	60.00	51.41 (51.19-51.57)	10235								
Armadillo	Dasypus novemcinctus	1.50	5.65	49.48 (49.35-49.60)	21565								
BushBaby	Otolemur garnettii	1.33	0.7715	50.17 (50.06-50.29)	18954								
Cat	Felis catus	0.63	4.75	50.20 (50.08-50.33)	18630	51.47 (50.85-51.90)	752	50.14 (50.00-50.29)	17878				
Chimp	Pan troglodytes	12.50	48.00	50.33 (50.18-50.46)	17831	51.69 (51.09-52.12)	617	50.27 (50.14-50.42)	17214	48.30 (47.96-48.59)	1938	50.38 (50.05-50.76)	1978
Cow	Bos taurus	1.00	755.00	48.93 (48.81-49.06)	19402	50.21 (49.71-50.62)	810	48.87 (48.72-49.00)	18592				
Devil	Sarcophilus harrisii	2.00	27.50	51.16 (51.04-51.27)	18008								
Dog	Canis familiaris	0.75	40.00	49.97 (49.85-50.08)	19227	50.54 (50.09-51.12)	802	49.94 (49.82-50.05)	18425				
Dolphin	Tursiops truncatus	10.25	230.00	50.11 (49.98-50.24)	15874								
Elephant	Loxodonta africana	11.00	4800.00	49.87 (49.75-49.99)	19410								
Ferret	Mustela putorius furo	0.50	1.50	49.96 (49.84-50.08)	19049								
Gibbon	Nomascus leucogenys	0.54	5.70	50.66 (50.52-50.76)	17806								
Gorilla	Gorilla gorilla	12.50	180.00	50.20 (50.09-50.33)	19476	50.88 (50.27-51.28)	817	50.18 (50.05-50.30)	18659				
GuineaPig	Cavia porcellus	0.25	0.9000	49.27 (49.17-49.40)	17948								
Hedgehog	Erinaceus europaeus	0.69	1.00	51.99 (51.89-52.11)	13759								
Horse	Equus caballus	2.58	1150.00	50.26 (50.13-50.37)	19727	51.95 (51.43-52.52)	831	50.15 (50.02-50.29)	18896				
Human	Homo sapiens	18.00	60.70	50.12 (49.99-50.26)	18027	50.92 (50.34-51.54)	758	50.08 (49.96-50.22)	17269	48.05 (47.71-48.46)	1903	49.57 (49.23-49.98)	2091
Hyrax	Procavia capensis	1.33	3.80	50.49 (50.37-50.61)	14986								
KangarooRat	Dipodomys ordii	0.17	0.0755	50.31 (50.19-50.44)	14629								
Macaque	Macaca mulatta	4.50	8.00	50.38 (50.26-50.48)	19868	51.20 (50.74-51.65)	861	50.33 (50.22-50.45)	19007				
Marmoset	Callithrix jacchus	1.37	330.00	50.23 (50.11-50.35)	19723	51.24 (50.60-51.72)	784	50.18 (50.07-50.31)	18939	48.93 (48.50-49.36)	1922	49.42 (49.10-49.70)	2430
Megabat	Pteropus vampyrus	2.00	0.8500	50.12 (49.97-50.25)	16234								
Microbat	Myotis lucifugus	0.58	0.0095	49.03 (48.90-49.17)	18908								
Mouse	Mus musculus	0.17	0.0210	50.41 (50.32-50.52)	19424	52.34 (51.93-52.74)	820	50.33 (50.23-50.41)	18604	49.56 (49.22-49.96)	1800	51.43 (51.01-51.64)	1346
MouseLemur	Microcebus murinus	0.67	0.0600	49.67 (49.50-49.82)	14771								
Opossum	Monodelphis domestica	0.50	0.1225	51.51 (51.40-51.62)	20541	51.87 (51.22-52.28)	480	51.50 (51.39-51.61)	20061				
Orangutan	Pongo abelii	15.88	60.00	50.26 (50.13-50.38)	18959	51.33 (50.57-51.79)	760	50.21 (50.06-50.34)	18199	48.22 (47.89-48.61)	2047	49.73 (49.40-50.09)	2407
Panda	Ailuropoda melanoleuca	5.75	102.50	49.89 (49.78-50.02)	18426								
Pig	Sus scrofa	0.75	200.00	49.27 (49.14-49.41)	20155	50.21 (49.73-50.74)	658	49.24 (49.09-49.37)	19497				
Pika	Ochotona princeps	0.98	0.1485	49.96 (49.84-50.09)	14559								
Platypus	Ornithorhynchus anatinus	1.75	1.65	50.03 (49.87-50.16)	17810	53.21 (52.36-53.74)	427	49.93 (49.79-50.07)	17383				
Rabbit	Oryctolagus cuniculus	0.67	2.00	49.77 (49.64-49.89)	18601	51.60 (51.08-52.08)	622	49.69 (49.57-49.82)	17979				
Rat	Rattus norvegicus	0.29	0.32	50.50 (50.40-50.57)	21994	52.02 (51.60-52.41)	834	50.43 (50.34-50.52)	21160				
Sheep	Ovis aries	2.00	110.00	49.53 (49.40-49.65)	19688	50.11 (49.74-50.68)	843	49.50 (49.37-49.64)	18845				
Shrew	Sorex araneus	0.79	0.0095	50.60 (50.43-50.76)	11073								
Sloth	Choloepus hoffmanni	3.75	6.00	51.81 (51.64-51.96)	10565								
Squirrel	Ictidomys tridecemlineatus	0.92	0.1250	49.52 (49.39-49.63)	18274								
Tarsier	Tarsius syrichta	1.42	0.1250	51.83 (51.69-51.95)	11813								
Tenrec	Echinops telfairi	0.07	0.2025	50.23 (50.11-50.40)	14625								
Treeshrew	Tupaia belangeri	0.38	0.1450	50.69 (50.51-50.86)	13746								
Wallaby	Macropus eugenii	1.38	6.55	51.70 (51.60-51.81)	13819								
