# Supplementary material for: Effective population size does not predict codon usage bias in mammals
Source: Ecol Evol. 2014 Sep 23;4(20):3887–900. doi: 10.1002/ece3.1249 (PMC4242573; doi:10.1002/ece3.1249)
Supplement: Supplementary file 6 — Appendix S5. The results of a phylogenetic generalized least squares correlation between ENC (not ENCp) and either generation time or body mass. All codons within an amino acid class were concatenated; because local base composition is nonsensical when combining codons across the genome, these tests were performed from ENC, not ENCp. [file ece30004-3887-SD5.pdf]

Appendix S5. The results of a phylogenetic generalized least squares correlation between ENC (not ENCp) and either generation time or body mass. All codons within an amino acid class were concatenated; because local base composition is nonsensical when combining codons across the genome, these tests were performed from ENC, not ENCp.

		generation time		body mass	
amino_acid	median_ENC	t-value	p	t-value	p
A	57.935	-0.247	0.806	-1.829	0.0751
C	60.911	0.377	0.708	0.104	0.918
D	60.848	1.131	0.265	0.290	0.7732
E	60.650	0.436	0.666	-0.715	0.4789
F	60.880	0.875	0.387	0.127	0.8994
G	59.887	0.678	0.502	0.061	0.9515
H	60.432	0.608	0.547	-0.711	0.4811
I	60.644	1.079	0.287	0.038	0.9697
K	60.727	0.912	0.367	0.022	0.9824
L (two-fold)	48.652	0.608	0.546	-0.068	0.9463
L (four-fold)	51.809	0.515	0.610	-0.554	0.5829
N	60.895	0.894	0.377	0.089	0.9295
P	59.115	-0.684	0.498	-0.057	0.9546
Q	57.804	0.261	0.796	-1.286	0.2059
R (four-fold)	53.689	0.444	0.659	-0.682	0.4993
R (two-fold)	49.018	4.810	0.000	-1.351	0.1845
S (four-fold)	53.371	-0.003	0.998	0.424	0.6742
S (two-fold)	48.749	0.553	0.583	-0.847	0.4023
T	58.906	0.478	0.636	-6.988	0
V	56.959	0.709	0.483	-0.468	0.6423
Y	60.730	1.0809	0.2864	0.1853	0.854
