# Supplementary material for: Effective population size does not predict codon usage bias in mammals
Source: Ecol Evol. 2014 Sep 23;4(20):3887–900. doi: 10.1002/ece3.1249 (PMC4242573; doi:10.1002/ece3.1249)
Supplement: Supplementary file 7 — Appendix S6. Results of ANCOVA's across species, testing the effect of X versus autosomal compartments (Chrom), the total number of coding base pairs (Exon), the total number of base pairs in introns between coding exons (Intron), and their interactions. [file ece30004-3887-SD6.pdf]

Appendix A6. Results of ANCOVA's across species, testing the effect X versus autosomal compartments (Chrom), the total number of coding base-pairs (Exon), the total number of base pairs in introns between coding exons (Intron), and their interactions. One transcript per gene was chosen randomly. In all species except opossum, X-linked genes had significantly lower codon usage bias compared to autosomal genes.

Organism	DF	Sum Square:	Mean Squares	F	Pr(>F)	Significance
Cat:						
Chrom (X vs. Auto)	1	637	637	17.328	3.16E-05	***
Exon (total coding bp)	1	6757	6757	183.919	2.00E-16	***
Intron (total bp)	1	19234	19234	523.554	2.00E-16	***
Interaction(Chrom*Exon)	1	49	49	1.323	0.25	
Interaction(Chrom*Intron)	1	3	3	0.083	0.774	
Interaction(Exon*Intron)	1	3401	3401	92.565	2.00E-16	***
interaction(Chrom*Exon*Intron)	1	0	0	0.001	0.976	
Residuals	18600	683311	37			
Chimp:						
Chrom (X vs. Auto)	1	635	635	16.908	3.94E-05	***
Exon (total coding bp)	1	7921	7921	210.962	2.00E-16	***
Intron (total bp)	1	14586	14586	388.475	2.00E-16	***
Interaction(Chrom*Exon)	1	18	18	0.472	0.492	
Interaction(Chrom*Intron)	1	28	28	0.74	0.39	
Interaction(Exon*Intron)	1	2646	2646	70.477	2.00E-16	***
interaction(Chrom*Exon*Intron)	1	0	0	0	0.985	
Residuals	17742	666139	38			
Cow:						
Chrom (X vs. Auto)	1	789	789	18.791	1.47E-05	***
Exon (total coding bp)	1	6418	6418	152.823	2.00E-16	***
Intron (total bp)	1	20574	20574	489.9	2.00E-16	***
Interaction(Chrom*Exon)	1	89	89	2.111	0.146	
Interaction(Chrom*Intron)	1	28	28	0.659	0.417	
Interaction(Exon*Intron)	1	2699	2699	64.264	1.15E-15	***
interaction(Chrom*Exon*Intron)	1	31	31	0.729	0.393	
Residuals	19369	813425	42			
Dog:						
Chrom (X vs. Auto)	1	237	237	5.886	0.0153	*
Exon (total coding bp)	1	9994	9994	247.983	<2e-16	***
Intron (total bp)	1	17160	17160	425.779	<2e-16	***
Interaction(Chrom*Exon)	1	21	21	0.528	0.4676	
Interaction(Chrom*Intron)	1	155	155	3.852	0.0497	*
Interaction(Exon*Intron)	1	3905	3905	96.879	<2e-16	***
interaction(Chrom*Exon*Intron)	1	1	1	0.015	0.9038	
Residuals	19202	773900	40			
Gorilla:						
Chrom (X vs. Auto)	1	148	148	3.81	0.051	.
Exon (total coding bp)	1	6588	6588	169.153	2.00E-16	***
Intron (total bp)	1	15303	15303	392.931	2.00E-16	***
Interaction(Chrom*Exon)	1	39	39	0.997	0.318	
Interaction(Chrom*Intron)	1	14	14	0.353	0.553	
Interaction(Exon*Intron)	1	1697	1697	43.566	4.20E-11	***
interaction(Chrom*Exon*Intron)	1	6	6	0.158	0.691	
Residuals	19436	756951	39			
Horse:						
Chrom (X vs. Auto)	1	1962	1962	52.037	5.64E-13	***
Exon (total coding bp)	1	4412	4412	117.003	2.00E-16	***
Intron (total bp)	1	10293	10293	272.956	2.00E-16	***

Interaction(Chrom*Exon)	1	37	37	0.973	0.3239	
Interaction(Chrom*Intron)	1	125	125	3.312	0.0688	.
Interaction(Exon*Intron)	1	1201	1201	31.858	1.68E-08	***
interaction(Chrom*Exon*Intron)	1	19	19	0.497	0.4809	
Residuals	19697	742756	38			
Human:						
Chrom (X vs. Auto)	1	434	434	10.946	0.00094	***
Exon (total coding bp)	1	7409	7409	186.733	2.00E-16	***
Intron (total bp)	1	16656	16656	419.811	2.00E-16	***
Interaction(Chrom*Exon)	1	0	0	0.006	0.93591	
Interaction(Chrom*Intron)	1	0	0	0.001	0.97856	
Interaction(Exon*Intron)	1	2644	2644	66.637	3.48E-16	***
interaction(Chrom*Exon*Intron)	1	30	30	0.75	0.38651	
Residuals	17963	712677	40			
Macaque:						
Chrom (X vs. Auto)	1	296	296	7.664	0.00564	**
Exon (total coding bp)	1	7477	7477	193.844	2.00E-16	***
Intron (total bp)	1	12162	12162	315.306	2.00E-16	***
Interaction(Chrom*Exon)	1	4	4	0.102	0.74988	
Interaction(Chrom*Intron)	1	198	198	5.138	0.02342	*
Interaction(Exon*Intron)	1	1984	1984	51.431	7.68E-13	***
interaction(Chrom*Exon*Intron)	1	34	34	0.88	0.34813	
Residuals	19811	764125	39			
Marmoset:						
Chrom (X vs. Auto)	1	528	528	13.07	0.000301	***
Exon (total coding bp)	1	8671	8671	214.712	2.00E-16	***
Intron (total bp)	1	15766	15766	390.395	2.00E-16	***
Interaction(Chrom*Exon)	1	61	61	1.512	0.218851	
Interaction(Chrom*Intron)	1	1	1	0.032	0.858999	
Interaction(Exon*Intron)	1	2108	2108	52.192	5.22E-13	***
interaction(Chrom*Exon*Intron)	1	33	33	0.824	0.364117	
Residuals	19665	794169	40			
Mouse:						
Chrom (X vs. Auto)	1	2245	2245	79.528	2.00E-16	***
Exon (total coding bp)	1	7682	7682	272.13	2.00E-16	***
Intron (total bp)	1	3946	3946	139.792	2.00E-16	***
Interaction(Chrom*Exon)	1	0	0	0.001	0.9734	
Interaction(Chrom*Intron)	1	86	86	3.059	0.08032	.
Interaction(Exon*Intron)	1	2458	2458	87.077	2.00E-16	***
interaction(Chrom*Exon*Intron)	1	239	239	8.459	0.00364	**
Residuals	19392	547409	28			
Opossum						
Chrom (X vs. Auto)	1	66	66	1.947	0.163	
Exon (total coding bp)	1	11655	11655	344.361	<2e-16	***
Intron (total bp)	1	9969	9969	294.548	<2e-16	***
Interaction(Chrom*Exon)	1	6	6	0.189	0.664	
Interaction(Chrom*Intron)	1	15	15	0.433	0.51	
Interaction(Exon*Intron)	1	2405	2405	71.062	<2e-16	***
interaction(Chrom*Exon*Intron)	1	1	1	0.025	0.874	
Residuals	20519	694451	34			
Orangutan:						
Chrom (X vs. Auto)	1	507	507	13.271	0.00027	***
Exon (total coding bp)	1	7471	7471	195.556	2.00E-16	***

Intron (total bp)	1	12485	12485	326.816	2.00E-16	***
Interaction(Chrom*Exon)	1	1	1	0.037	0.84668	
Interaction(Chrom*Intron)	1	73	73	1.904	0.16768	
Interaction(Exon*Intron)	1	2073	2073	54.257	1.83E-13	***
interaction(Chrom*Exon*Intron)	1	1	1	0.036	0.84935	
Residuals	18922	722882	38			
Pig:						
Chrom (X vs. Auto)	1	534	534	12.806	0.000346	***
Exon (total coding bp)	1	3381	3381	81.1	2.00E-16	***
Intron (total bp)	1	21802	21802	522.941	2.00E-16	***
Interaction(Chrom*Exon)	1	14	14	0.333	0.563815	
Interaction(Chrom*Intron)	1	15	15	0.368	0.54407	
Interaction(Exon*Intron)	1	1126	1126	27.001	2.05E-07	***
interaction(Chrom*Exon*Intron)	1	7	7	0.162	0.687525	
Residuals	20099	837944	42			
Platypus:						
Chrom (X vs. Auto)	1	2746	2746	61.394	4.94E-15	***
Exon (total coding bp)	1	6124	6124	136.903	2.00E-16	***
Intron (total bp)	1	24489	24489	547.452	2.00E-16	***
Interaction(Chrom*Exon)	1	228	228	5.088	0.0241	*
Interaction(Chrom*Intron)	1	169	169	3.778	0.0519	.
Interaction(Exon*Intron)	1	2556	2556	57.135	4.27E-14	***
interaction(Chrom*Exon*Intron)	1	36	36	0.802	0.3704	
Residuals	17758	794356	45			
Rabbit:						
Chrom (X vs. Auto)	1	1736	1736	45.524	1.55E-11	***
Exon (total coding bp)	1	8341	8341	218.722	2.00E-16	***
Intron (total bp)	1	16473	16473	431.96	2.00E-16	***
Interaction(Chrom*Exon)	1	126	126	3.305	0.0691	.
Interaction(Chrom*Intron)	1	51	51	1.325	0.2498	
Interaction(Exon*Intron)	1	3986	3986	104.516	2.00E-16	***
interaction(Chrom*Exon*Intron)	1	24	24	0.629	0.4276	
Residuals	18563	707898	38			
Rat:						
Chrom (X vs. Auto)	1	901	901	33.155	8.62E-09	***
Exon (total coding bp)	1	9962	9962	366.736	2.00E-16	***
Intron (total bp)	1	5452	5452	200.695	2.00E-16	***
Interaction(Chrom*Exon)	1	139	139	5.122	0.0236	*
Interaction(Chrom*Intron)	1	0	0	0.016	0.8984	
Interaction(Exon*Intron)	1	2517	2517	92.652	2.00E-16	***
interaction(Chrom*Exon*Intron)	1	0	0	0.002	0.9642	
Residuals	21959	596481	27			
Sheep:						
Chrom (X vs. Auto)	1	417	417	10.841	0.000994	***
Exon (total coding bp)	1	4473	4473	116.269	2.00E-16	***
Intron (total bp)	1	13554	13554	352.351	2.00E-16	***
Interaction(Chrom*Exon)	1	204	204	5.294	0.021407	*
Interaction(Chrom*Intron)	1	2	2	0.042	0.837312	
Interaction(Exon*Intron)	1	1495	1495	38.861	4.64E-10	***
interaction(Chrom*Exon*Intron)	1	1	1	0.022	0.883071	
Residuals	19656	756112	38			
