# Supplementary material for: Effective population size does not predict codon usage bias in mammals
Source: Ecol Evol. 2014 Sep 23;4(20):3887–900. doi: 10.1002/ece3.1249 (PMC4242573; doi:10.1002/ece3.1249)
Supplement: Supplementary file 9 — Appendix S7. Results of ANCOVA's across species, testing the effect of centromeric versus telomeric location (location), the total number of coding base pairs (Exon), the total number of base pairs in introns between coding exons (Intron), and their interactions. [file ece30004-3887-SD7.pdf]

Appendix S7. Results of ANCOVA's across species, testing the effect centromeric vs. telomeric location (location), the total number of coding base-pairs (Exon), the total number of base pairs in introns between coding exons (Intron), and their interactions. One transcript per gene was chosen randomly. In all species except opossum, X-linked genes had significantly lower codon usage bias compared to autosomal genes.

Organism	DF	Sum Squares	Mean Squares	F	Pr(>F)	Significance
Chimp:						
Location (Centro vs. Telo)	1	694	693.8	21.255	4.28E-06	***
Exon (total coding bp)	1	152	152.1	4.66	0.031	*
Intron (total bp)	1	1118	1117.7	34.239	5.70E-09	***
Interaction(Location*Exon)	1	48	47.6	1.457	0.228	
Interaction(Location*Intron)	1	15	14.7	0.451	0.502	
Interaction(Exon*Intron)	1	24	24.2	0.74	0.39	
interaction(Location*Exon*Intron)	1	13	12.6	0.385	0.535	
Residuals	1970	64309	32.6			
Human:						
Location (Centro vs. Telo)	1	1101	1100.9	32.261	1.55E-08	***
Exon (total coding bp)	1	386	386.1	11.313	0.000785	***
Intron (total bp)	1	1656	1656.2	48.532	4.42E-12	***
Interaction(Location*Exon)	1	2	2.1	0.063	0.802459	
Interaction(Location*Intron)	1	66	65.5	1.92	0.165991	
Interaction(Exon*Intron)	1	99	99.2	2.907	0.088375	.
interaction(Location*Exon*Intron)	1	4	4.3	0.126	0.722877	
Residuals	1957	66784	34.1			
Marmoset:						
Location (Centro vs. Telo)	1	49	49.3	1.287	0.256797	
Exon (total coding bp)	1	527	526.6	13.743	0.000215	***
Intron (total bp)	1	1478	1478.5	38.581	6.21E-10	***
Interaction(Location*Exon)	1	41	41.2	1.074	0.300065	
Interaction(Location*Intron)	1	6	5.9	0.155	0.694206	
Interaction(Exon*Intron)	1	34	34	0.888	0.346175	
interaction(Location*Exon*Intron)	1	8	8.2	0.214	0.643803	
Residuals	2320	88905	38.3			
Mouse:						
Location (Centro vs. Telo)	1	550	550.3	22.142	2.73E-06	***
Exon (total coding bp)	1	419	418.8	16.851	4.23E-05	***
Intron (total bp)	1	387	387.1	15.573	8.24E-05	***
Interaction(Location*Exon)	1	4	3.5	0.143	0.70578	
Interaction(Location*Intron)	1	35	35.4	1.424	0.23287	
Interaction(Exon*Intron)	1	169	168.7	6.789	0.00925	**
interaction(Location*Exon*Intron)	1	7	6.6	0.264	0.60713	
Residuals	1788	44439	24.9			
Orangutan:						
Location (Centro vs. Telo)	1	785	785.3	22.431	2.31E-06	***
Exon (total coding bp)	1	218	217.8	6.222	0.0127	*
Intron (total bp)	1	1025	1024.8	29.271	6.93E-08	***
Interaction(Location*Exon)	1	65	64.5	1.843	0.1747	
Interaction(Location*Intron)	1	144	143.8	4.108	0.0428	*
Interaction(Exon*Intron)	1	66	65.7	1.875	0.171	
interaction(Location*Exon*Intron)	1	0	0	0	0.9864	
Residuals	2341	81956	35			
