# Supplementary material for: Effective population size does not predict codon usage bias in mammals
Source: Ecol Evol. 2014 Sep 23;4(20):3887–900. doi: 10.1002/ece3.1249 (PMC4242573; doi:10.1002/ece3.1249)
Supplement: Supplementary file 11 — Appendix S8. Log10 ENCp versus log10 Ne across six mammal species. Bars represent 95% confidence intervals of medians estimated by bootstrapping the dataset 10,000 times. [file ece30004-3887-SD8.docx]

**Appendix S8.**
