# Supplementary material for: Effective population size does not predict codon usage bias in mammals
Source: Ecol Evol. 2014 Sep 23;4(20):3887–900. doi: 10.1002/ece3.1249 (PMC4242573; doi:10.1002/ece3.1249)
Supplement: Supplementary file 12 — Appendix S9. Point estimates and bootstrap ranges for Ne and ENCp for the six-species analysis. [file ece30004-3887-SD9.pdf]

Appendix S9. Point estimates and bootstrap ranges for Ne and ENCp

Species	Ne	Ne 95% bootstrap	ENCp	ENCp 95% bootstrap
Human	10,000	7,363-16,057	51.42	51.26-51.57
Chimp	25,000	18,545-40,046	51.23	51.05-51.39
M. *musculus*	60,000	45,575-95,542	51.27	51.14-51.39
M. *domesticus*	100,000	75,624-137,677	51.25	51.13-51.38
M. *castaneus*	220,000	156,067-271,423	51.23	51.12-51.35
Rabbit	780,000	572,651-1,060,260	50.39	50.23-50.58
