# Supplementary material for: Effective population size does not predict codon usage bias in mammals
Source: Ecol Evol. 2014 Sep 23;4(20):3887–900. doi: 10.1002/ece3.1249 (PMC4242573; doi:10.1002/ece3.1249)
Supplement: Supplementary file 14 — Appendix S10. The results of a phylogenetic generalized least squares correlation between ENC (not ENCp) and genetic estimates of Ne from six mammalian species per amino acid. All codons within an amino acid class were concatenated; because local base composition is meaningless when combining codons across the genome, these tests were performed with ENC, not ENCp. [file ece30004-3887-SD10.pdf]

Appendix S10. The results of a phylogenetic generalized least squares correlation between ENC (not ENCp) and genetic estimates of Ne from six mammalian species. All codons within an amino acid class were concatenated; because local base composition is nonsensical when combining codons across the genome, these tests were performed from ENC, not ENCp.

amino_acid	median_ENC	t-value	uncorrected p
A	57.9798	-4.98	0.0076
C	60.8893	-7.98	0.0013
D	60.8124	-9.38	0.0007
E	60.5010	-4.03	0.0157
F	60.7711	-4.49	0.0109
G	60.0071	-8.39	0.0011
H	60.3722	-10.63	4e-04
I	60.5498	-3.08	0.0369
K	60.2881	-7.18	0.002
L (four-fold)	51.8440	-9.29	7e-04
L (two-fold)	48.4875	-0.56	0.6073
N	60.6574	-1.69	0.1659
P	58.9117	**9.47**	7e-04
Q	57.4658	-4.52	0.0106
R (four-fold)	53.8870	-4.53	0.0106
R (two-fold)	48.9991	-8.83	9e-04
S (four-fold)	53.3000	0.70	0.5209
S (two-fold)	48.7191	-26.76	0
T	58.8219	**8.73**	9e-04
V	56.7733	-21.59	0
Y	60.5647	-7.70	0.0015
